# Novel and traditional anthropometric indices for identifying metabolic syndrome in non-overweight/obese adults

**DOI:** 10.1186/s12986-020-00536-x

**Published:** 2021-01-06

**Authors:** Lihong Wu, Wenhua Zhu, Qiaohua Qiao, Lijuan Huang, Yiqi Li, Liying Chen

**Affiliations:** Department of General Practice, Sir Run Run Shaw Hospital, School of Medicine, Zhejiang University, #3 East Qingchun Road, Hangzhou, 310016 Zhejiang China

**Keywords:** Anthropometric indice, Metabolic syndrome, Non-overweight/obese, Cardiovascular disease

## Abstract

**Background:**

Metabolic syndrome (MetS) in non-overweight/obese people is insidiously associated with cardiovascular disease. Novel anthropometric indices can reflect central obesity better than the traditional anthropometric indices. Therefore, we hypothesize that these newly developed anthropometric indices can better identify MetS in non-overweight/obese people than conventional indices.

**Methods:**

Cross-sectional data of sociodemographic, biochemical and anthropometric indices were collected from 2916 non-overweight/obese Chinese people. A body shape index (ABSI), body roundness index (BRI), waist-to-height ratio (WHtR), weight-adjusted-waist index (WWI) and abdominal volume index (AVI) were calculated. Partial correlation analysis was used to clarify the correlation between anthropometric indices and MetS variables. Binary logistic regression analysis was applied to assess the association between anthropometric indices and MetS and its components. Receiver-operating characteristic curve was used to identify the diagnostic ability of anthropometric indices for MetS and its components. The area under curve (AUC) difference between WHtR and each new anthropometric index was compared in pairs.

**Results:**

After adjusting for covariates, AVI had the optimal ability of identifying MetS (AUC: 0.743 for male, 0.819 for female) and the strongest correlation with high-density lipoprotein cholesterol (HDL-C) (coe: − 0.227 for male, − 0.207 for female) and the highest odds rations (OR) with low HDL-C group (male: OR = 1.37, female: OR = 1.55). The WHtR was comparable to BRI in assessing MetS (AUC: 0.739 for male, 0.817 for female). WHtR or BRI could also well identify hypertension (AUC: 0.602 for male, 0.688 for female) and dysglycemia (AUC: 0.669 for male, 0.713 for female) and female’s high triglyceride level (AUC 0.712). The recognition ability of the two was equivalent. The ability of ABSI and WWI to identify MetS was weak.

**Conclusions:**

AVI is the optimal anthropometric indices to identify MetS in non-overweight/obese Chinese adults. BRI and WHtR can also be considered as discriminators, while ABSI and WWI are weak discriminators. WHtR is easy to measure. So, it is recommended as an early preliminary screening method for the MetS in non-overweight/obese people.

## Introduction

MetS is a cluster of cardiometabolic risk, including dysglycemia, elevated blood pressure, raised triglyceride levels, low high-density lipoprotein cholesterol levels, and central adiposity. Previous studies have found that at least one in four adults have MetS worldwide [1]. Since the risk of arteriosclerotic cardiovascular disease (ASCVD) in people with MetS is twice than that of non-MetS, people with MetS may account for half of all ASCVD [2]. In addition, MetS increased the likelihood of type 2 diabetes fivefold [2]. Although the incidence of MetS has been closely associated with obesity [3], metabolic disorders are often hidden in non-overweight/obese people [4–9], especially in Asians [8]. The China National Diabetes and Metabolic Disorders Study [9] found that metabolic disorders accounted for 46.2% of people with normal weight.

Visceral fat plays a critical role in MetS pathogenesis [10]. Since visceral fat is mainly concentrated in the abdomen, for a long time, people have simply screened and managed visceral obesity through central obesity anthropometric indices, such as waist circumference (WC), body mass index (BMI) and WHtR [11]. However, some studies found that these indices provide limited information about fat distribution. BMI is a rough indices of obesity since individuals with similar BMI may present with different degree of fatness [12]. Whereas WC is unclear what extent depending on body size [13]. WHtR has been shown to be superior to BMI and WC in screening central obesity [14–16]. Studies has shown that it was well correlated with a variety of cardiometabolic components [17], which was not supported by others [18]. Therefore, it is necessary to design more suitable anthropometric indices that combine body shape and disease prediction ability to measure central obesity.

BRI, ABSI and AVI were all novel indices of body geometry and good predictors of visceral fat [19–21]. BRI was optimal in identifying the metabolic components and arteriosclerosis of overweight/obese people [22–24]. ABSI has been shown to be associated with visceral fat, carotid atherosclerosis [25] and obesity-related death risk independent of BMI and WC [20]. AVI indirectly reflected visceral fat content through assessing of the entire abdominal volume. It is closely related to impaired glucose tolerance (IGT) and diabetes mellitus (DM) [21], and has good predictive ability for MetS [26]. WWI was a unique obesity index which was found by Park et al. [27] in a study of 465,629 South Koreans in 2018. It had excellent predictive power for cardiometabolic disease, CVD and all-cause mortality risk. These four indices have a strong ability to identify MetS in terms of body shape and disease identification ability. However, whether these new anthropometric indicators are superior to traditional anthropometric indicators and whether they can better identify MetS in non-overweight/obese people needs further research.

Thus, the current study was designed to compare conventional and novel indices for identifying MetS in non-overweight/obese Chinese adults. Meanwhile, we tried to screen out the most suitable anthropometric indices for MetS in non-overweight/obese Chinese adults and the optimal cut-off point based on different genders.

## Methods

### Study population

In the present study, 4790 participants who underwent routine check-ups were recruited from June 2018 to June 2019 at the check-ups center of Sir Run Run Shaw Hospital, affiliated with Medical College of Zhejiang University. All participants were Han ethnicity and came from more than 30 provinces in mainland China. Each participant finished a standard questionnaire which included information on age, body weight, disease history, medication history, and family history of cardiometabolic diseases and CVD. Inclusion criteria were BMI < 24 kg/m^2^ and age between 18 and 75 years. Exclusion criteria were: (1) a history of corticosteroid or hormone therapy in the past 6 months; (2) those who have received weight loss program or lost weight ≥ 5% in the past 12 months; (3) a history of cardiovascular and cerebrovascular disease, heart failure, arrhythmia, malignant tumor, edema, viral hepatitis, cirrhosis, hepatic and renal insufficiency, thyroid dysfunction, skeletal malformation or amputation or dependence on a wheelchair or other mobile assistance device; (4) more than 14 units of alcohol per week for males (1 unit = 14 g of alcohol) and more than 10 units per week for females; (5) pregnancy. Finally, 2916 subjects (1215 males and 1701 females) were recruited. After fasting for one night, all subjects completed blood sample collection, anthropometric measurements and a short questionnaire.

The study was approved by the Ethics Committee of Sir Run Run Shaw Hospital, affiliated with Medical College of Zhejiang University. All participants provided written informed consent before taking part in the study.

### Anthropometric measurements

Body height and weight were measured on the digital scale with light clothing and no shoes with the accuracy 0.1 cm and 0.1 kg, respectively. WC was measured at the end of a normal exhalation by placing a tape measure on the horizontal surface between the lower rib and the iliac crest, at the accuracy of 0.1 cm. Hip circumference was measured at the maximum extension of the hip bone. WHtR was calculated as WC (cm)/height (cm). BMI was obtained by dividing the participant’s weight (kg) by a square of height (m^2^). BRI, WWI, AVI and ABSI were calculated according to the following formula [19–21, 27]:

$$\begin{aligned} {\text{BRI}} & = 364.2 - 365.5 \times \sqrt {1 - \left( {\frac{{\left( {{\text{WC}}/2\uppi } \right)^{2} }}{{\left( {0.5 \times {\text{height}}} \right)^{2} }}} \right)} \\ {\text{AVI}} & = \frac{{2 \times ({\text{waist}})^{2} + 0.7 \times \left( {{\text{wasit}} - {\text{hip}}} \right)^{2} }}{1000} \\ {\text{WWI}} & = \frac{{{\text{WC}}}}{{\sqrt {{\text{weight}}} }} \\ {\text{ABSI}} & = \frac{{{\text{WC}}}}{{{\text{BMI}}^{2/3} {\text{height}}^{1/2} }} \\ \end{aligned}$$

### Clinical and biochemical tests

Blood pressure was monitored using a standard sphygmomanometer (OMRON 705IT). After the subjects sat for 10 min, the researchers took two blood pressure records from their right arms. The average value of the two data was used for statistical analysis. The ARCHITECT C16000 chemical analyzer was used to measure fasting blood glucose (FBG), uric acid (UA), C-reactive protein (CRP), total cholesterol (TCHO), low-density lipoprotein cholesterol (LDL-C), HDL-C and triglyceride (TG) in a standard laboratory according to standard procedures.

### Definition of MetS

In the present study, MetS was diagnosed in accordance with the definition of the Chinese guidelines for the Prevention and Treatment of dyslipidemia in adults (2016 revision) [28]. The participants were categorized as MetS when they met three or more of the following components: (1) abdominal obesity: WC ≥ 90 cm for males or ≥ 85 cm for females; (2) Hyperglycemia: fasting blood glucose ≥ 6.10 mmol/L (110 mg/dL) or blood glucose ≥ 7.80 mmol/L (140 mg/dL) 2 h after glucose load and/or diabetes had been diagnosed and treated; (3) Hypertension: blood pressure ≥ 130/85 mmHg and/or hypertension has been diagnosed and treated; (4) Fasting TG ≥ 1.7 mmol/L (150 mg/dL); (5) Fasting HDL-C < 1.0 mmol/L (40 mg/dL).

### Statistical analysis

SPSS 23.0 (IBM) was used for statistical analysis and MedCalc Version19.1 for ROC curve comparison. Data was described as mean and standard deviation for continuous variables and as frequencies and percentage for categorical variables. The study subjects were characterized by independent sample *t* test, nonparametric test (continuous variable) or χ^2^ test (categorical variables) according to gender. Partial correlation analysis was applied to evaluate the correlation between various anthropometric indexes and metabolic variables such as systolic blood pressure (SBP), diastolic blood pressure (DBP), TG, HDL-C and FBG. Binary logistic regression analysis assessed the relationship between anthropometric indicators and MetS and its components. Age and CRP were adjusted by partial correlation analysis and binary logistic regression analysis, and z-scores of anthropometric indices were used. The ROC and AUC were used to assess the ability of five anthropometric indices to identify MetS and its components. Method described by Hanley and McNeil [29] was used to assess AUC differences in MetS among BRI, WWI, AVI, ABSI and WHtR. Finally, the optimal cut-off values of five anthropometric indicators for MetS identification were determined. *p* < 0.05 was considered statistically significant.

## Results

### The demographic characteristics, clinical and anthropometric data of the study population

A total of 2916 non-overweight/obese subjects (1215 for male and 1701 for female) participated in the study. The prevalence of MetS was 4.9% (7.6% in males and 3.0% in females), and the prevalence of dyslipidemia (21.65%) and hypertension (22.7%) was higher. Physical measurement indicators (height, weight, BMI, WC, WHtR, BRI, ABSI, WWI, AVI), clinical indicators (SBP, DBP, FBG, TC, TG, LDL-C, CRP, UA) and the incidence of MetS and its components were significantly higher in males than in females (except HDL-C) (Table 1).

**Table 1:** Characteristics of the study participants according to gender among non-overweight/obese adults

Variables	Total (n = 2916)	Male (n = 1215)	Female (n = 1701)	*p* value
Age(years)	47.04 ± 10.38	47.91 ± 10.70	46.41 ± 10.11	< 0.001
Education				
Low (< 9 y)	711(24.4%)	254(20.9%)	457(26.9%)	< 0.001
Middle (9–12 y)	1035(35.5%)	439(36.1%)	596(35.0%)	< 0.001
High (> 12 y)	1170(40.1%)	522(43.0%)	648(38.1%)	< 0.001
Height (cm)	164.06 ± 7.96	170.78 ± 5.89	159.26 ± 5.36	< 0.001
Weight (kg)	57.68 ± 7.73	63.64 ± 6.42	53.39 ± 5.42	< 0.001
WC (cm)	77.33 ± 7.42	82.08 ± 5.87	73.95 ± 6.49	< 0.001
WHtR	0.47 ± 0.04	0.48 ± 0.03	0.46 ± 0.04	< 0.001
BMI (kg/m^2^)	21.36 ± 1.77	21.80 ± 1.64	21.05 ± 1.78	< 0.001
AVI	12.22 ± 2.21	13.64 ± 1.85	11.20 ± 1.87	< 0.001
ABSI	0.0784 ± 0.004	0.0805 ± 0.0038	0.0769 ± 0.0049	< 0.001
WWI	10.20 ± 0.64	10.30 ± 0.54	10.13 ± 0.70	< 0.001
BRI	2.87 ± 0.71	3.03 ± 0.61	2.76 ± 0.75	< 0.001
SBP (mmHg)	116.55 ± 15.97	119.18 ± 14.46	114.68 ± 16.72	< 0.001
DBP (mmHg)	69.42 ± 10.48	72.20 ± 10.30	67.44 ± 10.16	< 0.001
FBG (mmol/L)	5.18 ± 0.90	5.31 ± 1.08	5.09 ± 0.74	< 0.001
TC (mmol/L)	4.77 ± 0.94	4.82 ± 0.96	4.74 ± 0.93	0.038
TG (mmol/L)	1.38 ± 1.18	1.63 ± 1.45	1.21 ± 0.91	< 0.001
HDL-C (mmol/L)	1.34 ± 0.33	1.23 ± 0.31	1.43 ± 0.32	< 0.001
LDL-C (mmol/L)	2.62 ± 0.72	2.70 ± 0.73	2.57 ± 0.72	< 0.001
CRP (mg/L)	1.17 ± 3.04	1.43 ± 3.91	0.99 ± 2.19	< 0.001
UA (umol/L)	316.74 ± 79.67	374.34 ± 71.46	275.60 ± 56.31	< 0.001
High BP level (n, %)	663(22.7%)	307(25.3%)	356(20.9%)	0.006
Abdominal obesity (n, %)	195(6.7%)	101(8.3%)	94(5.5%)	0.003
Dysglycemia (n, %)	188(6.4%)	109(9.0%)	79(4.6%)	< 0.001
High TG level (n, %)	630(21.65%)	381(31.4%)	249(14.6%)	< 0.001
Low HDL-C level (n, %)	389(13.3%)	284(23.4%)	105(6.2%)	< 0.001
MetS (n,%)	143(4.9%)	92(7.6%)	51 (3.0%)	< 0.001

*p* value, means the differences between groups according to gender among non-overweight/obese adults

*WC* waist circumference, *WtHR* waist-to-height ratio, BMI body mass index, *AVI* abdominal volume index, *ABSI* a body shape index, *WWI* weight adjusted waist index, *BRI* body roundness index, *SBP* systolic blood pressure, *DBP* diastolic blood pressure, *FBG* fasting blood glucose, *TC* total cholesterol, *TG* triglyceride, *HDL-C* HDL cholesterol, *LDL-C* LDL cholesterol, *CRP* C-reactive protein, *UA* uric acid, *MetS* metabolic syndrome

### Partial correlation between different anthropometric indices and metabolic variables

After adjusting for age, UA and CRP, most anthropometric measures were significantly correlated with metabolic variables. The correlation between BRI, WHtR, AVI and metabolic variables was stronger than that between ABSI and WWI. AVI had the strongest negative correlation with HDL-C (coe: − 0.192 for males and females). ABSI showed the weakest correlation (Table 2).

**Table 2:** Partial correlations between anthropometric indices with metabolic variables

	WHtR		BRI		WWI		AVI		ABSI	
	coe	*p*	coe	*p*	coe	*p*	coe	*p*	coe	*p*
Male										
SBP	0.099	0.001	**0.101**	< 0.001	0.029	0.307	0.1	0.001	− 0.008	0.769
DBP	**0.159**	< 0.001	**0.159**	< 0.001	0.103	< 0.001	0.158	< 0.001	0.068	0.019
FBG	**0.137**	< 0.001	0.136	< 0.001	0.096	< 0.001	0.122	< 0.001	0.06	0.036
TG	**0.177**	< 0.001	0.176	< 0.001	0.146	< 0.001	0.162	< 0.001	0.112	< 0.001
HDL-C	− 0.184	< 0.001	− 0.182	< 0.001	− 0.134	< 0.001	− **0.192**	< 0.001	− 0.111	< 0.001
LDL-C	**0.103**	< 0.001	0.099	0.001	0.062	0.031	0.082	0.004	0.026	0.374
Female										
SBP	0.111	< 0.001	0.116	< 0.001	0.088	< 0.001	**0.117**	< 0.001	0.076	0.002
DBP	0.086	< 0.001	0.09	< 0.001	0.068	0.005	**0.101**	< 0.001	0.063	0.01
FBG	0.141	< 0.001	**0.15**	< 0.001	0.14	< 0.001	0.134	< 0.001	0.122	< 0.001
TG	0.205	< 0.001	**0.218**	< 0.001	0.203	< 0.001	0.216	< 0.001	0.183	< 0.001
HDL-C	− 0.172	< 0.001	− 0.168	< 0.001	− 0.102	< 0.001	− **0.192**	< 0.001	− 0.078	0.001
LDL-C	**0.08**	0.001	0.077	0.001	0.075	0.002	0.056	0.022	0.053	0.028

The partial correlation is adjusted for age, UA and CRP. Bold indicates the strongest related anthropometric indices for different metabolic variables

*WtHR* waist-to-height ratio, *BRI* body roundness index, *WWI* weight adjusted waist index, *AVI* abdominal volume index, *ABSI* a body shape index, *SBP* systolic blood pressure, *DBP* diastolic blood pressure, *FBG* fasting blood glucose, *TG* triglyceride, *HDL-C* high-density lipoprotein cholesterol, *LDL-C* low-density lipoprotein cholesterol

### Binary logistic regression analysis of anthropometric indicators and MetS and its components

The OR and 95% confidence interval (CI) were analyzed using anthropometric Z-scores after controlling age, UA and CRP. WHtR, AVI, BRI were independently correlated with MetS and its components. These five anthropometric indexes had the high OR for MetS, among which WHtR had the highest dominance ratio in females (OR = 2.812, *p* < 0.001) and AVI had the highest dominance ratio in males (OR = 2.45, *p* < 0.001). The OR of ABSI for MetS was the lowest (males OR = 1.607, *p* < 0.001; females OR = 2.068, *p* < 0.001) (Table 3).

**Table 3:** The correlation between MetS and its components with anthropometric indexes

	High BP		Dysglycemia		Low HDL-C		High TG		MetS	
	OR	95% CI	OR	95% CI	OR	95% CI	OR	95% CI	OR	95% CI
Male										
WHtR	**1.263**^**#**^	1.087–1.467	1.561*	1.230–1.981	1.303^#^	1.12–1.516	1.727*	1.487–2.006	2.357*	1.788–3.106
BRI	1.254^#^	1.082–1.453	1.522*	1.212–1.912	1.287^#^	1.11–1.493	1.696*	1.465–1.963	2.235*	1.728–2.890
WWI	1.089^^^	0.94–1.262	1.309^#^	1.046–1.639	1.226^#^	1.056–1.422	1.523*	1.319–1.759	1.738*	1.362–2.217
AVI	1.205^#^	1.046–1.388	1.441*	1.162–1.788	1.331*	1.153–1.535	1.585*	1.38–1.82	2.45*	1.898–3.163
ABSI	0.981^^^	0.851–1.130	1.164^^^	0.941–1.441	1.208^#^	1.047–1.393	1.343*	1.173–1.537	1.607*	1.276–2.205
Female										
WHtR	**1.253**^**#**^	1.076–1.460	**1.46**^**#**^	1.125–1.893	1.442*	1.149–1.811	1.634*	1.384–1.929	2.812*	2.003–3.948
BRI	1.251^#^	1.079–1.451	1.432^#^	1.126–1.821	1.414^#^	1.139–1.756	1.578*	1.346–1.851	2.565*	1.881–3.498
WWI	1.197^#^	1.030–1.391	1.426^#^	1.114–1.827	1.212^^^	0.973–1.509	1.462*	1.249–1.713	2.171*	1.62–2.91
AVI	1.213^#^	1.056–1.393	1.392^#^	1.115–1.737	1.49*	1.22–1.819	1.472*	1.269–1.707	2.768*	2.052–3.733
ABSI	1.142^^^	0.991–1.315	1.362^#^	1.077–1.723	1.156^^^	0.938–1.425	1.313*	1.132–1.523	2.068*	1.567–2.728

The bold indicates the highest value of odds ratio among the indices. The binary logistic regression analyses are adjusted for age, UA, and CRP

*WtHR* waist-to-height ratio, *BRI* body roundness index, *WWI* weight adjusted waist index, *AVI* abdominal volume index, *ABSI* a body shape index, *BP* blood pressure, *TG* triglyceride, *HDL-C* HDL cholesterol, *MetS* metabolic syndrome, *OR* odds ratio, *95% CI* 95% confidence interval

^*^p < 0.001; ^#^*p* < 0.05; ^^^*p* > 0.05

### The diagnostic ability of anthropometric indicators for MetS and its components

**Fig. 1:**
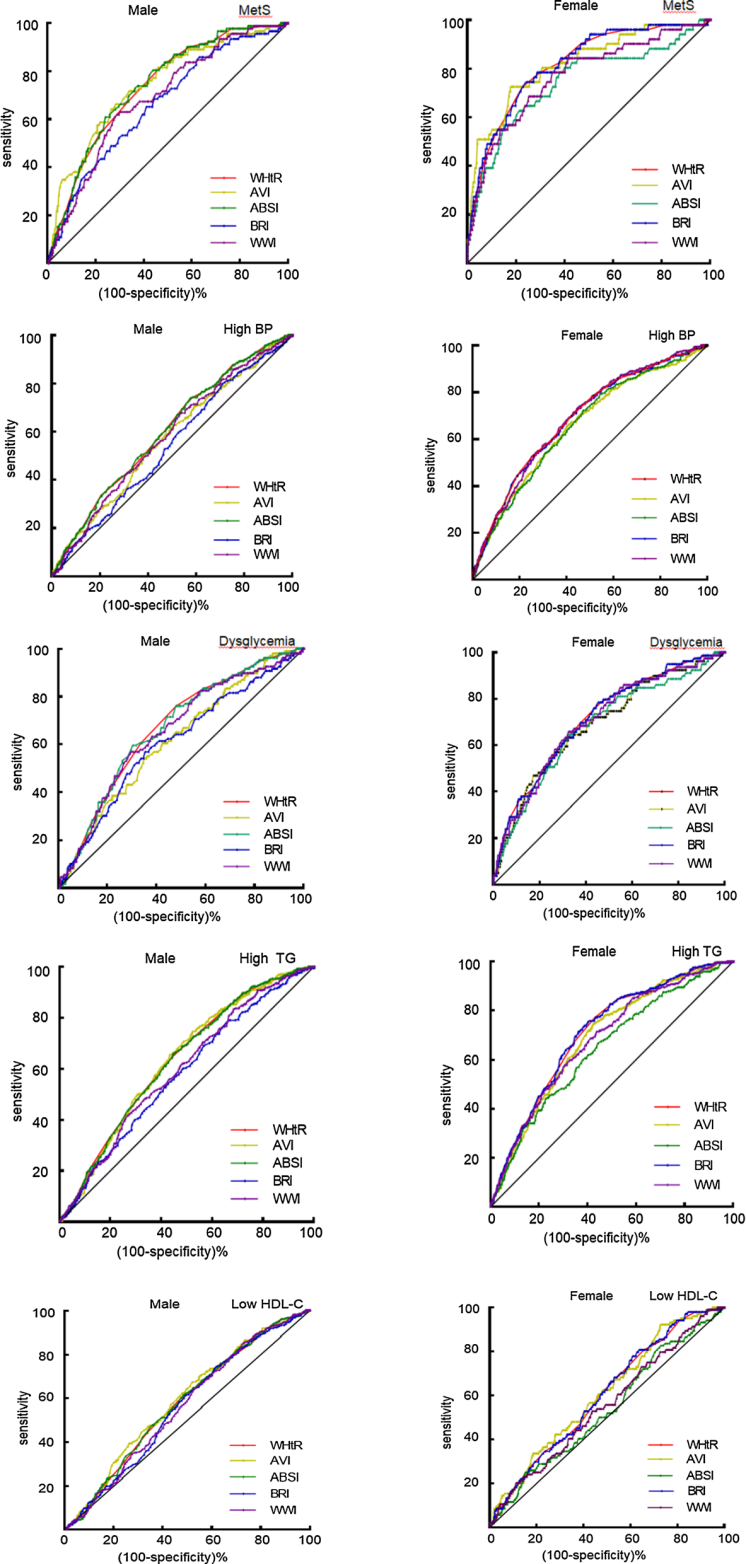
The discriminative power of the anthropometric indices for MetS and its components. *WtHR* waist-to-height ratio, *BRI* body roundness index, *WWI* weight adjusted waist index, *AVI* abdominal volume index, *ABSI* a body shape index, *BP* blood pressure, *TG* triglyceride, *HDL-C* HDL cholesterol, *MetS* metabolic syndrome

As shown in Talbe 4 and Fig. 1, AVI had the best ability to identify MetS (AUC: 0.743 for male, 0.819 for female) and low HDL-C (AUC: 0.591 for male, 0.614 for female). The recognition of WHtR and BRI for MetS were similar (AUC: 0.739 for males and 0.817 for females).

### The differences in ROC curves of anthropometric indices for MetS identification

There were no statistically significant differences between AVI with WHtR and BRI, while there were significant differences between WHtR with ABSI and WWI. The ability of WHtR to identify MetS was comparable to that of BRI and AVI, but significantly superior to that of ABSI and WWI (Tables 4, 5).

**Table 4:** AUC and its 95% CI for each body index according to MetS components

	High BP		Dysglycemia		Low HDL-C		High TG		MetS	
	AUC	95% CI	AUC	95% CI	AUC	95% CI	AUC	95% CI	AUC	95% CI
Male										
WHtR	0.602*	0.574–0.630	0.669*	0.641–0.695	0.579*	0.543–0.616	0.633*	0.605–0.660	0.739*	0.691–0.787
BRI	0.602*	0.574–0.630	0.669*	0.641–0.695	0.579*	0.543–0.616	0.633*	0.605–0.660	0.739*	0.691–0.787
WWI	0.581*	0.552–0.609	0.655*	0.627–0.682	0.563*	0.527–0.599	0.598*	0.565–0.632	0.689*	0.636–0.742
AVI	0.571*	0.543–0.599	0.617*	0.589–0.644	0.591*	0.555–0.628	0.634*	0.606–0.666	0.743*	0.690–0.796
ABSI	0.540^#^	0.512–0.569	0.608*	0.580–0.665	0.561^#^	0.525–0.598	0.579*	0.550–0.607	0.663*	0.606–0.719
Female										
WHtR	0.688*	0.666–0.710	0.713*	0.691–0.734	0.605*	0.581–0.628	0.712*	0.690–0.733	0.817*	0.798–0.835
BRI	0.688*	0.666–0.710	0.713*	0.691–0.734	0.605*	0.581–0.628	0.712*	0.690–0.733	0.817*	0.798–0.835
WWI	0.688*	0.665–0.710	0.705*	0.682–0.726	0.556*	0.532–0.580	0.684*	0.661–0.706	0.768*	0.748–0.788
AVI	0.659*	0.636–0.682	0.692*	0.669–0.713	0.614*	0.590–0.637	0.690*	0.667–0.712	0.819*	0.800–0.837
ABSI	0.660*	0.637–0.682	0.682*	0.660–0.704	0.541^^^	0.484–0.598	0.647*	0.623–0.669	0.744*	0.723–0.765

Bold indicates the maximum

*WtHR* waist-to-height ratio, *BRI* body roundness index, *WWI* weight adjusted waist index, *AVI* abdominal volume index, *ABSI* a body shape index, *BP* blood pressure, *TG* triglyceride, *HDL-C* HDL cholesterol, *MetS* metabolic syndrome

^*^p < 0.001; ^#^*p* < 0.05; ^^^*p* > 0.05

**Table 5:** Pairwise comparison for ROC curves for the identification of MetS

	WHtR -BRI	WHtR -ABSI	WHtR -AVI	WHtR -WWI	BRI -ABSI	BRI -AVI	BRI -WWI	ABSI -AVI	ABSI -WWI	AVI -WWI
Male										
Diff. AUC	0.005	0.072	0.009	0.046	0.077	0.003	0.051	0.081	0.026	0.055
SE	0.003	0.02	0.02	0.02	0.02	0.02	0.01	0.02	0.01	0.02
*p* value	0.079	**0.003**	0.64	**0.003**	**< 0.001**	0.84	**< 0.001**	**< 0.001**	0.06	**0.026**
Female										
Diff. AUC	0.001	0.072	0.003	0.048	0.073	0.002	0.049	0.075	0.024	0.051
SE	0.002	0.03	0.02	0.02	0.03	0.02	0.02	0.03	0.02	0.03
*p* value	0.70	**< 0.001**	0.88	**0.006**	**0.007**	0.93	**0.004**	**< 0.001**	0.12	**0.049**

*P* values marked in bold are significant

*AUC* area under curve, *SE* standard error, *WtHR* waist-to-height ratio, *BRI* body roundness index, *WWI* weight adjusted waist index, *AVI* abdominal volume index, *ABS* a body shape index

### The optimal cutoff value of sex-based anthropometric indices for the identification of MetS

The optimal cut-off value of each anthropometric index for evaluating MetS included WHtR (0.571 for males and 0.49 for females), BRI (3.41 for males, 3.24 for females), AVI (14.47 for males, 12.83 for females). When the optimal cutoff point value was obtained, AVI showed the highest sensitivity (0.717) to identify MetS in men and the highest specificity (0.823) in women. WHtR had the highest specificity (0.76) for screening MetS in men and the highest sensitivity for identifying women (0.766) (Table 6).

**Table 6:** The optimal cut-off values and its sensitivity and specificity for identification of MetS

	Male			Female		
	Cut-off	Sens	Spec	Cut-off	Sens	Spec
WHtR	0.571	0.609	**0.760**	0.49	**0.766**	0.745
BRI	3.41	0.609	**0.760**	3.24	0.745	0.766
WWI	10.57	0.609	0.724	10.56	0.686	0.75
AVI	14.47	**0.717**	0.663	12.83	0.725	**0.823**
ABSI	0.0813	0.652	0.591	0.0806	0.627	0.796

Bold indicates the maximum specificity/sensitivity

*WtHR* waist-to-height ratio, *BRI* body roundness index, *WWI* weight adjusted waist index, *AVI* abdominal volume index, *ABSI* a body shape index

## Discussion

To date, there have been only a few studies of anthropometric indicators of MetS recognition in non-overweight/obese people, and the results are not consistent with the latest anthropometric indicators. This cross-sectional study innovatively compared the identification ability of the novel central obesity index with traditional indicators for non-overweight/obese individuals with a large sample size and a larger population coverage. The results demonstrate that the novel anthropometric index can identify MetS in non-overweight/obese people. Among them, AVI had the best ability to identify MetS and low HDL-C in different genders.

A growing number of studies showed that cardiometabolic disease often occurs in people with normal weight [4–6, 9]. Visceral adipose tissue (VAT) accumulation is a major cause [30]. Clinicians often use anthropometric indices which reflect VAT to screen MetS in large populations. For a long time, BMI combined with WC has been extensively used to assess central obesity. But both two predicted all-cause mortality in the opposite way in some cases [31]. The paradox occurs when the distinction of body fat is not made to predict cardiometabolic risk [12]. Therefore, the assessment of central obesity and the prediction of cardiometabolic disease by BMI combined with WC are limited. In this study, people with BMI < 24 kg/m^2^ were taken as the study subjects, and MetS was taken as the disease, avoiding the assessment of BMI combined with WC.

WHtR, another widely used traditional anthropometric index, is superior to BMI and WC in the assessment of central obesity [14–16, 32]. As a simple and effective anthropometric index, it has been recommended by many scholars as a screening tool for cardiometabolic risk factors. Even in people with normal BMI and/or WC, WHtR can effectively identify cardiometabolic disease [33, 34]. However, meta-analysis based on Embase and Medline databases showed that WHtR was not superior to other anthropometric indicators in distinguishing MetS and other cardiometabolic factor [18]. Moreover, these studies did not compare WHtR with sundry new anthropometric indexes such as WWI and AVI, and it was still uncertain whether the WHtR in non-overweight/obese people is the optimal anthropometric index to screen MetS.

Ulike the traditional anthropometric index, the new anthropometric index mostly started from the geometric model of the human body, reflected the VAT of the body in a three-dimensional way. Based on waist and hip circumference, AVI calculated the entire abdominal volume from symphysis of pubis to xiphoid appendix [21], theoretically including abdominal free fat and adipose tissue volumes, which are the main distribution areas of visceral fat [35]. VAT has fewer insulin receptors distributed on the cell surface, decreased insulin receptor substrate protein-1 expression, and reduced insulin receptor affinity. Therefore, VAT becomes less sensitive to insulin and has lower sugar uptake and utilization [36]. In addition, free fatty acid produced by VAT lipolysis affects insulin signaling pathway, reduces the sensitivity of liver and skeletal muscle to insulin, inhibits glucose uptake and oxidation, and aggravates glucose regulation disorders [37]. Therefore, AVI demonstrated excellent predictive power for IGT and DM by fully evaluating VAT [21]. Previous study found that the predictive ability of AVI for MetS was better than other new anthropometric indicators and traditional indicators [38, 39], and was the strongest predictor [1], which was similar to the results of this study. In this study, AVI’s ability to identify MetS in non-overweight/obese people was lower than that in Spanish adolescents (AUC: 0.831 for males, 0.867 for females) [38], but higher than that in northern Iran (AUC: 0.72 for males, 0.73 for females). This was related to race, age and BMI range of the study population [40, 41].

This study found that BRI and WHtR had similar ability to screen MetS, which were similar to the results of previous studies [23, 42]. WHtR reflects central obesity through simple numerical comparison, which overcomes the influence of height on VAT [43]. However, BRI is a human body ellipse model, which evaluates body fat rate and VAT according to roundness and eccentricity [19] and quantifies individual body shape in an independent manner of height. Although the two anthropometric indicators have different principles, they are all derived from WC and height. VAT assessment is also based on WC and abdominal fat volume. Therefore, BRI and WHtR had the similar recognition capability for MetS, but they had no statistical difference with AVI. Compared with AVI and BRI, WHtR is easier to obtain and more suitable for early preliminary screening in a large population.

WWI is a recently developed unique obesity index based on weight and WC [27], which can predict the incidence and mortality of obesity-related diseases with a linear trend, avoiding the U-shaped relationship between BMI and CVD mortality. Until now, there have been no studies on the recognition capability of WWI for MetS and no studies on the prognosis of metabolic diseases in non-overweight/obese people. Our study found that WWI was less able to identify MetS in non-overweight/obese people than WHtR. It was speculated that WWI was based on body weight and WC and it could not distinguish fat distribution and body weight composition, so the fat content was underestimated. Since height was not taken into account, WWI usage may underestimate the VAT of short subjects and overestimate the VAT of tall subjects, thus misleading the diagnosis of central obesity and failing to predict the prevalence of MetS. ABSI is another body shape index based on WC, weight and height [20]. At a given height and weight, high ABSI means WC is higher than expected, which is a good indicator of central obesity. ABSI changes the limitations of WC as a BMI dependent index [20]. Studies have shown that ABSI can identify visceral obesity and sarcopenic obesity in overweight/obese adults with T2DM [44]. However, this study showed that, similar to WWI, ABSI was also weak in predicting MetS in non-overweight/obese people. Similar findings have been found in other studies [45, 46]. It is speculated that although these two indexes are new obesity indexes, both the establishment and verification of the prediction model are to make up for the deficiency of BMI in the prediction of obesity-related mortality risk, and the identification of MetS may affect its diagnostic efficacy. To realize the screening of MetS or cardiometabolic disease, further formulation and a large sample population studies are needed.

In addition, anthropometric indices were better at identifying MetS in females than in males after adjusting for confounding factors. This cannot be explained by conventional wisdom, which males have more visceral fat and females have more subcutaneous adipose tissue [40]. This may be related to the large number of female subjects. Further studies on the predictive power of novel anthropometric indicators for MetS of different genders are needed.

Currently, the novel anthropometric indicators have shown advantages in various fields. They are worthy of clinical and public health promotion for their ability to predict obesity-related diseases and deaths at an earlier stage, but new longitudinal studies are needed in a broader population to further explore their predictive power.

Our study has several limitations. First, this was a cross-sectional study and it cannot reflect causality. Second, information on the lifestyle and drug therapy of the participants was not obtained in the study. These indices were all derived from WC. These may confound the relationship between anthropometric indices and MetS. Third, the participants were volunteers who were more concerned about their health and who might have a history of CVD or family history. Finally, we did not measure 2 h postprandial blood glucose, which may lead to under diagnosis in some diabetes patients.

## Conclusions

Despite the limitations of the study, our research results showed that AVI was the optimal anthropometric index for the identification of MetS in non-overweight/obese Chinese adults. BRI and WHtR can also be taken into account as discriminators, while ABSI and WWI are weak discriminators. WHtR was simple and easy to measure. It was recommended as an early primary screening method for MetS in non-overweight/obese people.

## Data Availability

The datasets used and analyzed during the current study available from the corresponding author on reasonable request.

## Abbreviations

WC: Waist circumference
WtHR: Waist-to-height ratio
BMI: Body mass index
AVI: Abdominal volume index
ABSI: A body shape index
WWI: Weight adjusted waist index
BRI: Body roundness index
SBP: Systolic blood pressure
DBP: Diastolic blood pressure
FBG: Fasting blood glucose
TC: Total cholesterol
TG: Triglyceride
HDL-C: High-density lipoprotein cholesterol
LDL-C: Low-density lipoprotein cholesterol
CRP: C-reactive protein
UA: Uric acid
MetS: Metabolic syndrome
CVD: Cardiovascular disease
ASCVD: Arteriosclerotic cardiovascular disease
IGT: Impaired glucose tolerance
VAT: Visceral adipose tissue
DM: Diabetes mellitus
OR: Odds ratio
CI: Confidence interval
ROC: Receiver-operating characteristic
AUC: Area under the curve
